# Bilateral ischemic optic neuropathy after transurethral prostatic resection: a case report

**DOI:** 10.1186/1471-2415-6-32

**Published:** 2006-10-11

**Authors:** Luis M Sadaba, Alfredo Garcia-Layana, Miguel J Maldonado, Jose M Berian

**Affiliations:** 1 Ophthalmology Department, Clínica Universitaria de Navarra, Universidad de Navarra, Pamplona (Navarra), Spain; 2 Urology Department, Clínica Universitaria de Navarra, Universidad de Navarra, Pamplona (Navarra), Spain

## Abstract

**Background:**

Nonarteritic ischemic optic neuropathy affects the anterior portion of the optic nerve and is characterized by sudden, painless visual loss. The affected eye has a relative afferent pupillary defect. The typical funduscopic appearance includes optic disc edema, with associated nerve fiber layer hemorrhage. Risk factors include advanced age, systemic hypertension, nocturnal hypotension, diabetes mellitus, and a small cup-to-disc ratio. Bilateral presentation is rare. Postoperative optic neuropathy has been associated with nonocular surgery; risk factors include a combination of prolonged surgical times, acute systemic hypotension, anemia due to blood loss, or prone positioning. We report for the first time a patient with bilateral, simultaneous anterior ischemic optic neuropathy after elective transurethral prostatic resection.

**Case presentation:**

A 66-year old man underwent surgery for benign prostatic hyperplasia. The preoperative blood pressure was 140/85 mmHg, hemoglobin 15.9 g/dL, and hematocrit 48.6%. Two hours postoperatively, the blood pressure, hemoglobin, and hematocrit dropped dramatically. One day later, transient horizontal diplopia developed. Funduscopy showed a congenitally small cup-to-disc ratio without papillary edema. Other ocular findings were unremarkable. By 4 days postoperatively, sudden and painless amaurosis bilaterally developed when the patient awoke with nausea and vomiting. Visual acuity was no light perception bilaterally. The optic discs were swollen with small hemorrhages. Scans of the head and orbits and electrolyte levels were normal. There were no responses on visual evoked potentials bilaterally. The blood pressure was 90/50 mm Hg, the hemoglobin 7.0 g/dL, and the hematocrit 22.9%, necessitating infusion of three units of packed red blood cells. The blood pressure, hematocrit, and hemoglobin increased to normal levels. Three months later the visual acuity remained no light perception. The pupils were unreactive and there was marked optic disc atrophy bilaterally.

**Conclusion:**

Bilateral and simultaneous acute ischemic optic neuropathy may be a rare but devastating surgical complication. The combination of anemia and hypotension may increase the risk of anterior ischemic optic neuropathy postoperatively after transurethral prostatic resection.

## Background

Nonarteritic ischemic optic neuropathy (NAION) is an ischemic process of the anterior portion of the optic nerve. The typical presentation is sudden and painless visual loss. The affected eye has a relative afferent pupillary defect, unless there is bilateral symmetric optic nerve disease. The typical funduscopic appearance includes sectoral or generalized optic disc edema, which may be mildly pale or hyperemic, with associated nerve fiber layer hemorrhage. The presence of optic disc edema is a requisite for a diagnosis of NAION. This process has been associated with various risk factors, including advanced age, systemic hypertension, nocturnal hypotension, diabetes mellitus, and optic disc morphology (small cup-to-disc ratio). The occurrence of bilateral simultaneous NAION is exceedingly uncommon [1].

Postoperative optic neuropathy has been associated with many types of nonocular surgeries [2-4]. Risk factors include a combination of prolonged surgical times, acute systemic hypotension, anemia due to blood loss, or prone positioning [4]. When optic neuropathy occurs after blood loss, simultaneous involvement of both eyes is not unusual [5].

We report a patient with bilateral and simultaneous anterior ischemic optic neuropathy (AION) after elective transurethral prostatic resection (TPR). To the best of our knowledge such a complication after TPR has not been previously reported

## Case presentation

**Figure 1:**
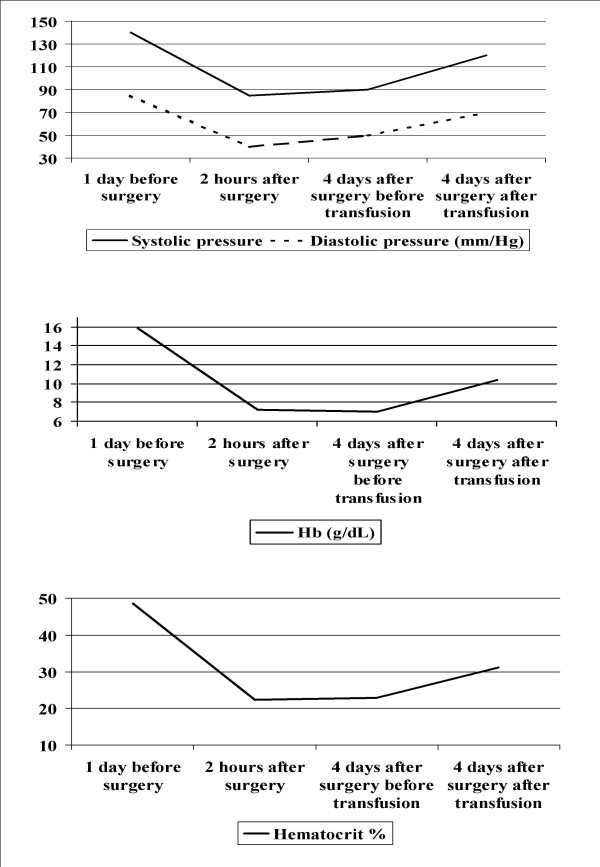
Top: Blood pressure. Middle: Hemoglobin levels. Bottom: Hematocrit levels.

A 66-year old man underwent elective TPR for benign prostatic hyperplasia. The preoperative blood pressure was 140/85 mmHg, hemoglobin 15.9 g/dL, and hematocrit 48.6%. Surgery was performed with glycine irrigation. Two hours after surgery, the blood pressure was 85/40 mm Hg, hemoglobin 7.2 g/dL, and hematocrit 22.3% (figure 1). One day later, he reported transient horizontal diplopia when he looked to the right, and the oculomotility was normal. The visual acuity was 20/20 bilaterally, pupillary reflexes were normal, and the intraocular pressure by applanation tonometry was 15 mmHg bilaterally. Slit-lamp examination showed a minimal cataract. Funduscopy showed a congenitally anomalous optic nerve head with small cup-to-disc ratio and no papillary edema.

**Figure 2:**
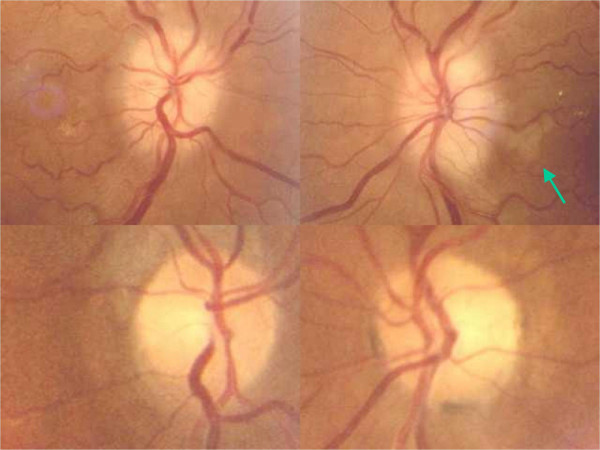
Top: Funduscopic examination revealed pale and swollen discs with small hemorrhages on both optic discs. Top right, an area of retinal infarction can be observed immediately off the disc (arrow). Bottom. Three months later, both optic discs are pale and atrophic.

Two days after surgery he had transient episodes of blurred vision bilaterally and no diplopia. Four days after surgery, upon awakening he experienced sudden and painless amaurosis in both eyes with nausea and vomiting. On examination, he had no light perception bilaterally. Funduscopic examination revealed that both optic discs were swollen with small hemorrhages. Further, an area of parapapillary retinal infarction was present in the left eye (figure 2). Computerized axial tomography and magnetic resonance imaging scans of the head and orbits were performed and the results were normal. Electrolyte levels were within normal limits (sodium 140 mEq/L, potassium 3.8 mEq/L, chloride 102 mEq/L). There were no responses on visual evoked potentials in both eyes. The rest of the ocular examination was unremarkable. The blood pressure was 90/50 mm Hg, the hemoglobin 7.0 g/dL, and the hematocrit 22.9% (figure 1), necessitating infusion of three units of packed red blood cells. The blood pressure increased to 120/70, the hematocrit to 31.1%, and the hemoglobin to 10.4 g/dL (figure 1).

He was examined 3 months later and the visual acuity in both eyes remained no light perception. The pupils did not react to light and the funduscopic examination showed marked optic disc atrophy in both eyes (figure 2).

## Conclusion

Postoperative visual loss may be the result of several different mechanisms, including anterior and posterior ischemic optic neuropathy, cortical blindness, retinal artery occlusion, and ophthalmic venous obstruction [2].

Rarely, ischemic optic neuropathy may present as an early and extremely serious perioperative complication that can involve both optic nerves simultaneously [5]. Stevens et al. suggested that prolonged controlled hypotension and intraoperative anemia could increase the risk of ischemic optic neuropathy in patients with predisposing factors [6].

Another common entity after transurethral prostatic resection is the TURP (transurethral resection of the prostate) syndrome [7]. Endoscopy of the genitourinary tract requires the use of an irrigating fluid containing glycine 1.5% and exposes patients to adverse events related to absorption of the irrigating fluid, referred to as the TURP syndrome. This syndrome is caused by dilutional hyponatremia (serum sodium, < 125 mEq/L) and is characterized by mental confusion, nausea, vomiting, hypertension, bradycardia, and visual disturbances [7]. In our case, the serum sodium was between 140 and 141 mEq/L and the TURP syndrome was then not considered a diagnosis.

Our patient presented with AION 4 days after TPR, preceded by episodes of transient diplopia and blurred vision. Bilateral AION associated with an area of retinal infarction immediately off the left optic disc (figure 2) could suggest a vasculitic mechanism, such as giant cell arteritis. The erythrocyte sedimentation rate and C-reactive protein were not elevated and no jaw claudication or scalp tenderness was found. In addition, there was no temporal artery abnormality (tenderness or reduced pulsation).

A case of AION after rotator cuff surgery was reported recently [3]. The patient had hyperlipidaemia and anomalous optic nerves. The only risk factor during surgery was prolonged hypotension. The mean arterial pressure decreased by 41.6% [3]. In our case, the combination of anomalous nerves, hypotension, and anemia could be the risk factors for AION.

Bilateral and simultaneous acute ischemic optic neuropathy may be a rare but devastating surgical complication. Medical personnel should be aware that the combination of anemia and hypotension may increase the risk of AION in the postoperative period after TPR, and that transient episodes of diplopia and blurred vision after TPR may herald that process.

## Competing interests

The author(s) declare that they have no competing interests.

## Contributions of the authors

**LMS **participated in the design of the study, and prepared the first draft of the manuscript. **AGL **and **MJM **participated in the analysis and interpretation of the data. **JMB **examined the patient and designed the study. All authors contributed to the editing and revising of the manuscript and all authors have read and approved the final version.

## Pre-publication history

The pre-publication history for this paper can be accessed here:


